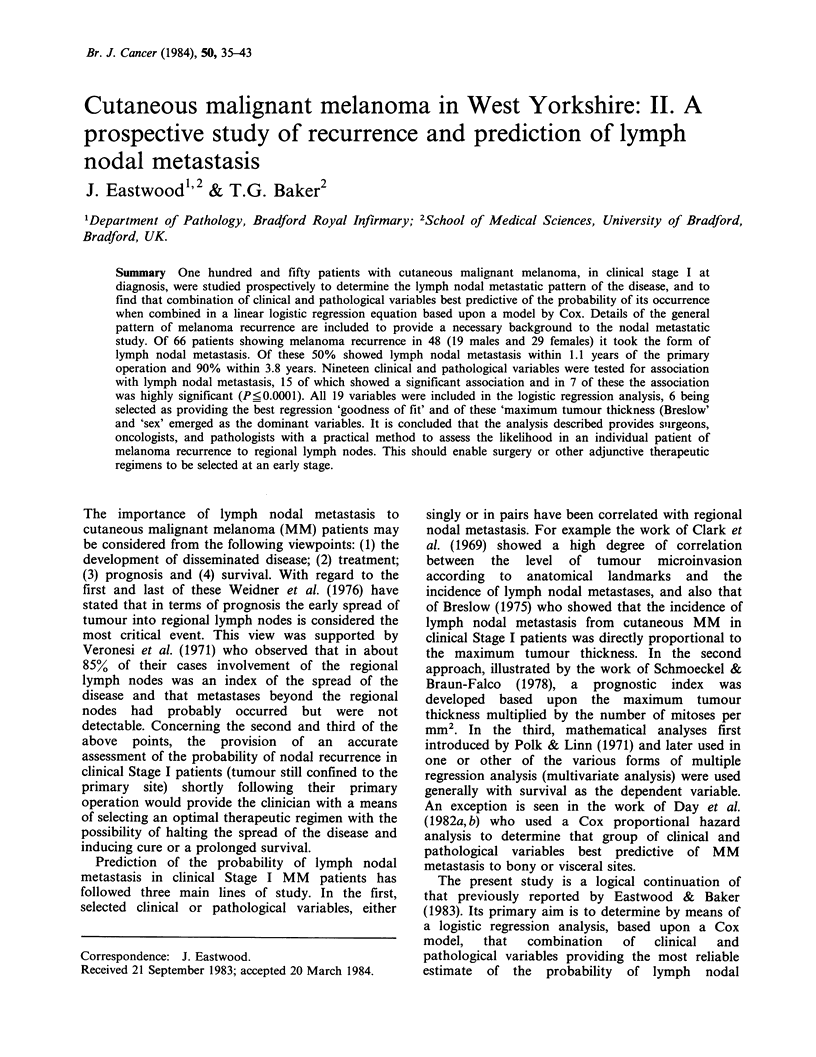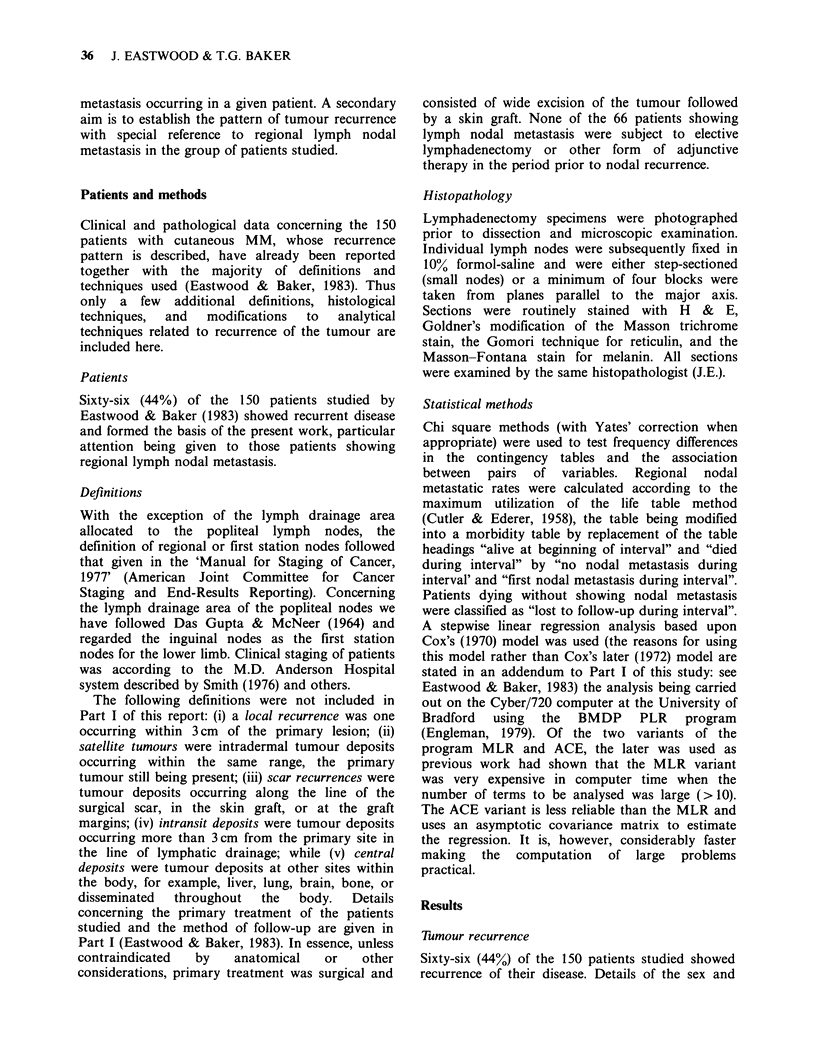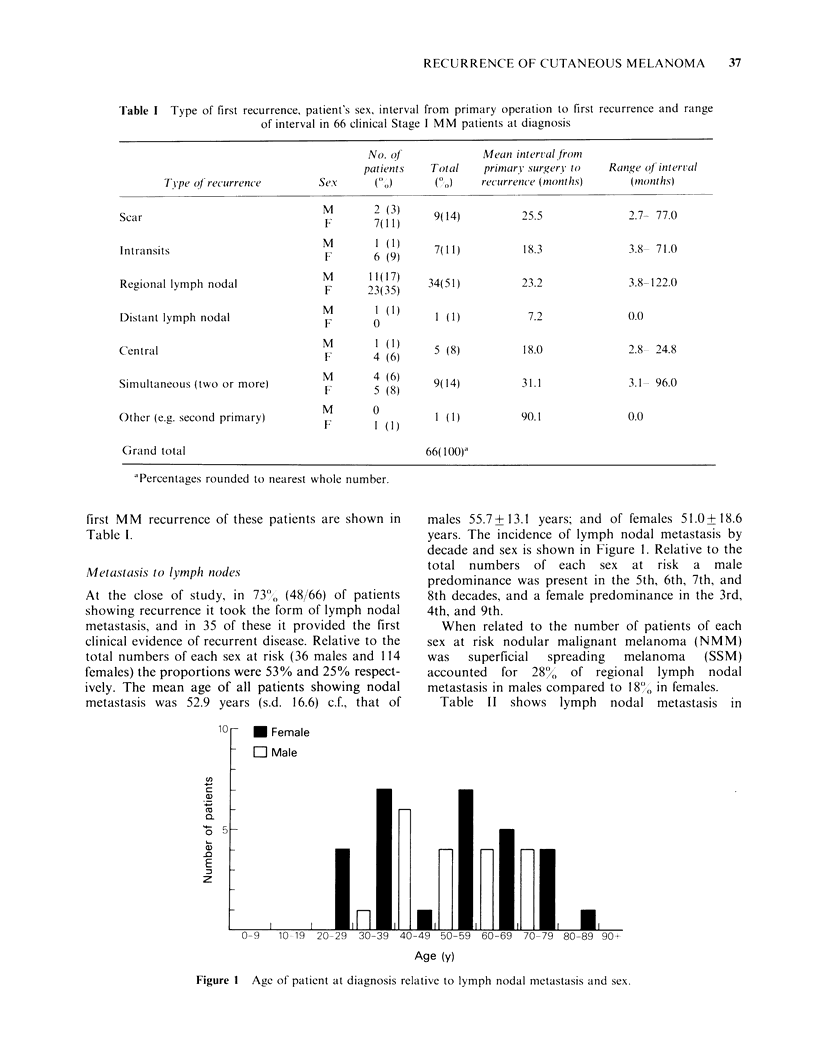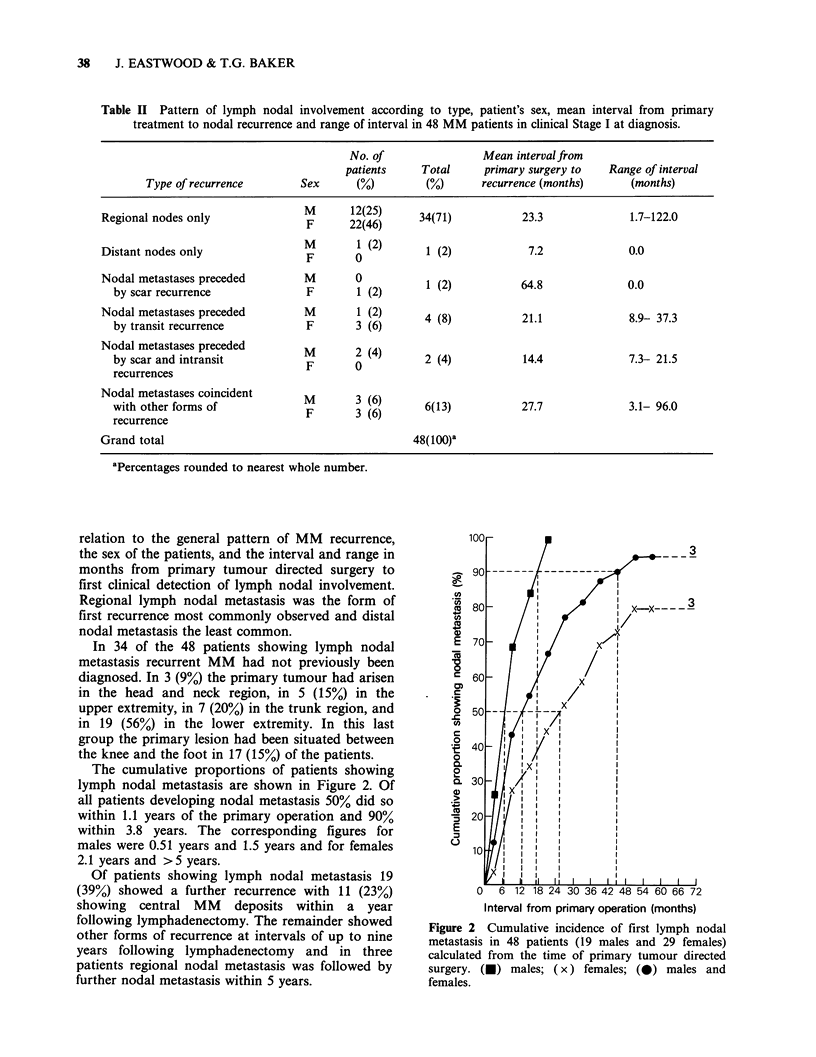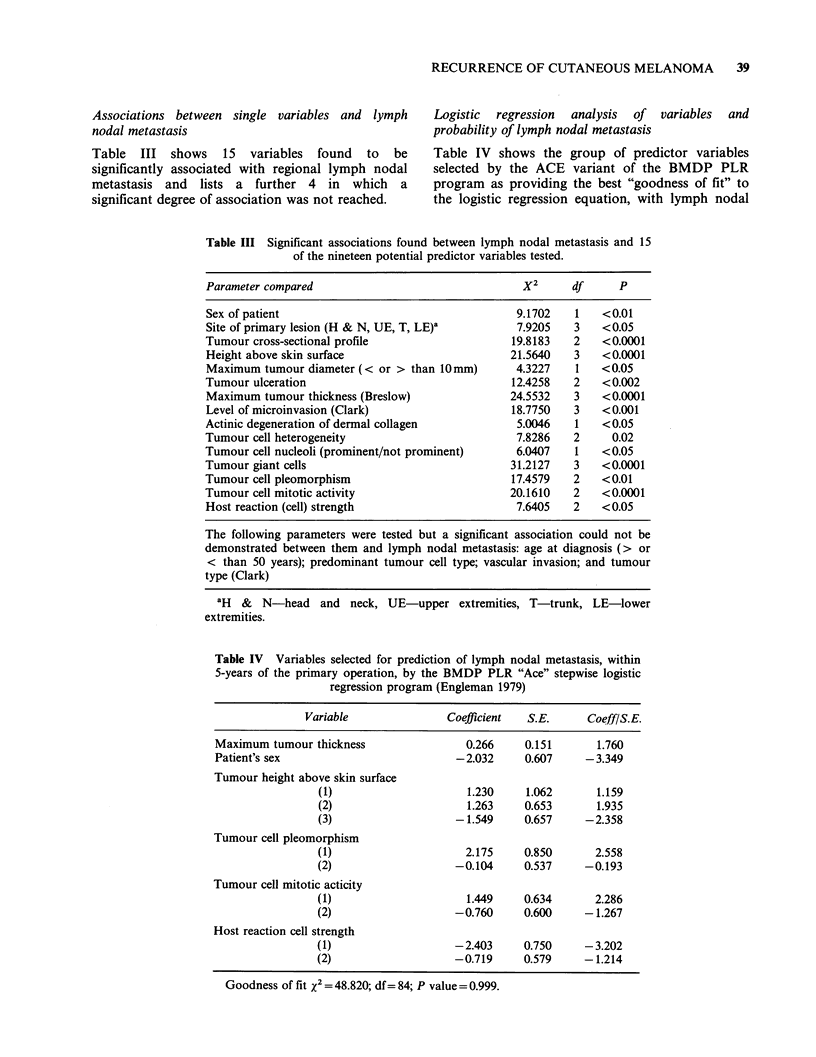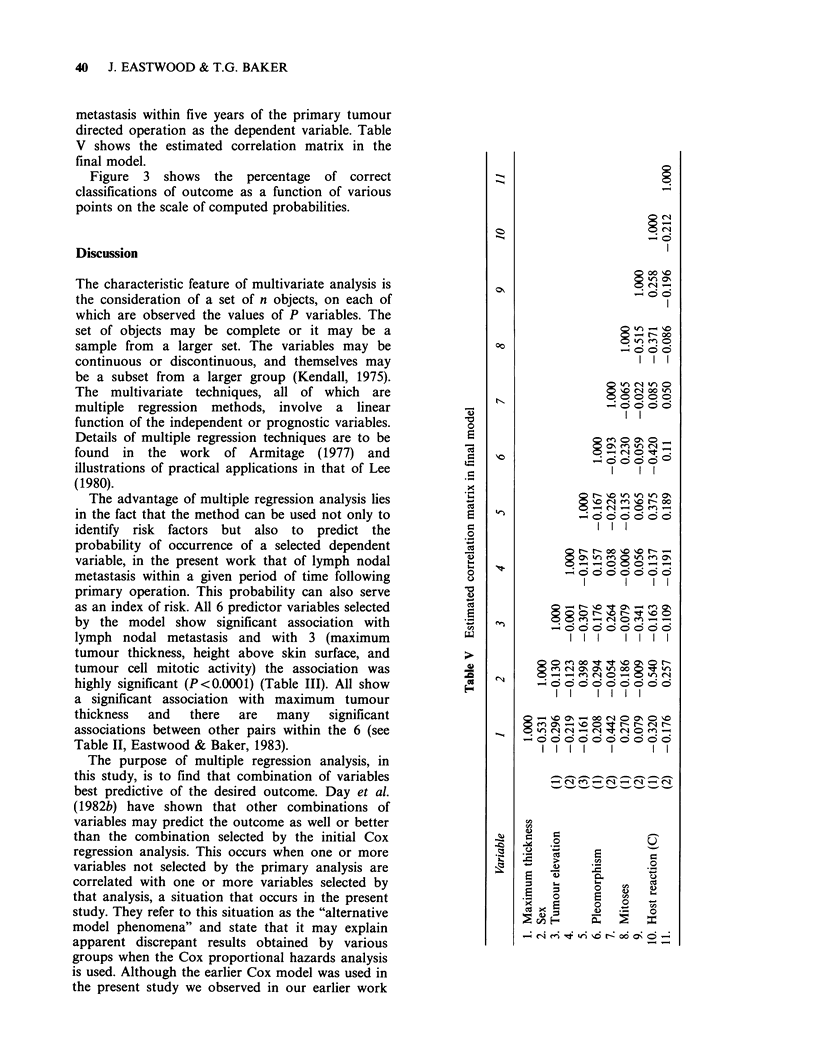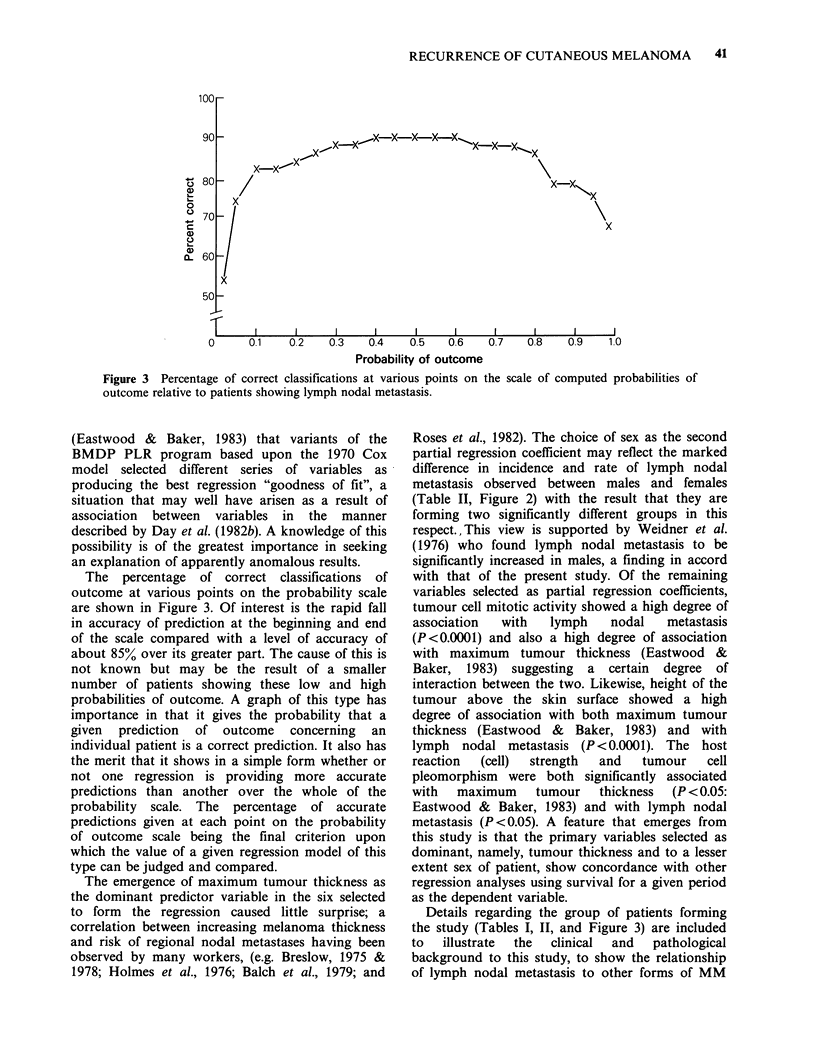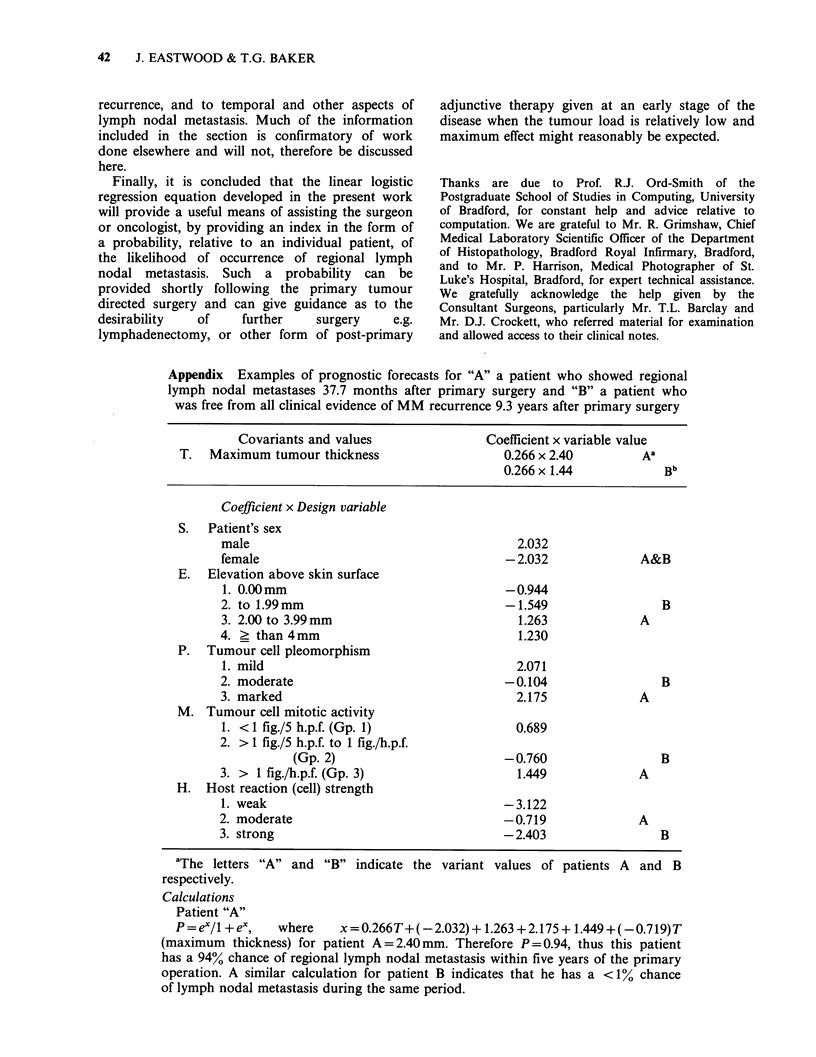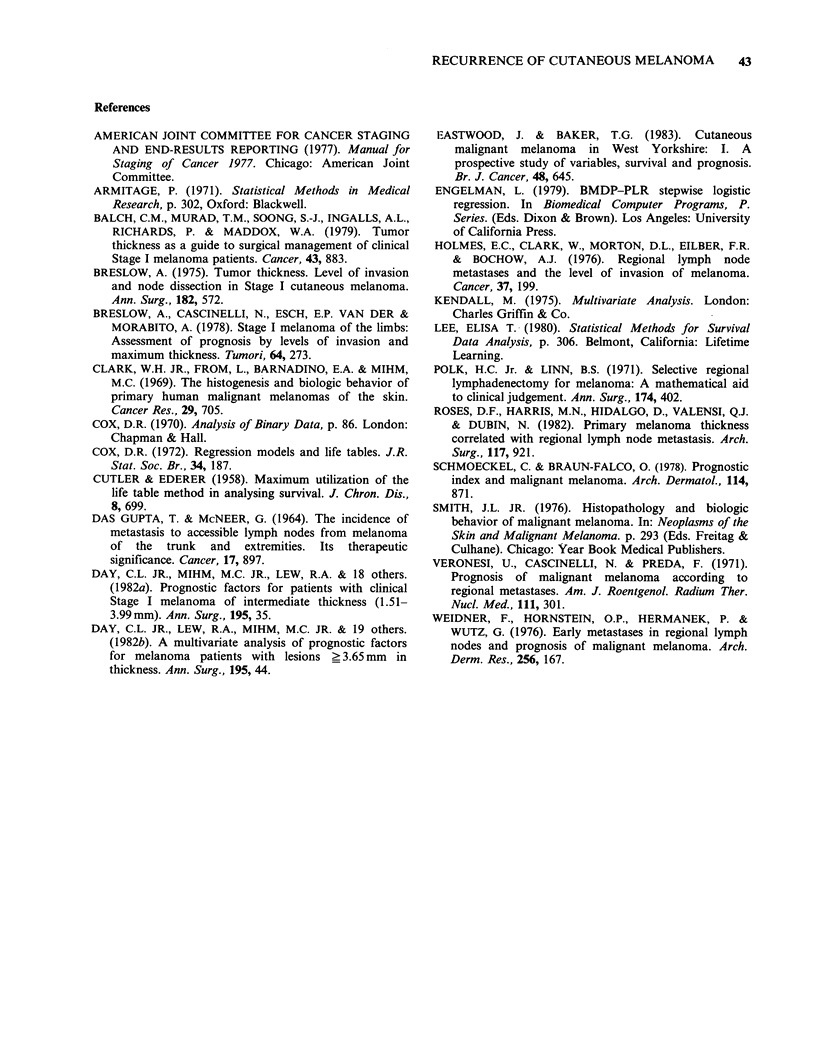# Cutaneous malignant melanoma in West Yorkshire: II. A prospective study of recurrence and prediction of lymph nodal metastasis.

**DOI:** 10.1038/bjc.1984.137

**Published:** 1984-07

**Authors:** J. Eastwood, T. G. Baker

## Abstract

One hundred and fifty patients with cutaneous malignant melanoma, in clinical stage I at diagnosis, were studied prospectively to determine the lymph nodal metastatic pattern of the disease, and to find that combination of clinical and pathological variables best predictive of the probability of its occurrence when combined in a linear logistic regression equation based upon a model by Cox. Details of the general pattern of melanoma recurrence are included to provide a necessary background to the nodal metastatic study. Of 66 patients showing melanoma recurrence in 48 (19 males and 29 females) it took the form of lymph nodal metastasis. Of these 50% showed lymph nodal metastasis within 1.1 years of the primary operation and 90% within 3.8 years. Nineteen clinical and pathological variables were tested for association with lymph nodal metastasis, 15 of which showed a significant association and in 7 of these the association was highly significant (P less than or equal to 0.0001). All 19 variables were included in the logistic regression analysis, 6 being selected as providing the best regression 'goodness of fit' and of these 'maximum tumour thickness (Breslow' and 'sex' emerged as the dominant variables. It is concluded that the analysis described provides surgeons, oncologists, and pathologists with a practical method to assess the likelihood in an individual patient of melanoma recurrence to regional lymph nodes. This should enable surgery or other adjunctive therapeutic regimens to be selected at an early stage.


*Br. J. Cancer* (1984), **50**, 35–43

### Cutaneous malignant melanoma in West Yorkshire: II. A prospective study of recurrence and prediction of lymph nodal metastasis

J. Eastwood[1,2] & T.G. Baker[2]

[1]*Department of Pathology, Bradford Royal Infirmary;* [2]*School of Medical Sciences, University of Bradford, Bradford, UK.*

**Summary** One hundred and fifty patients with cutaneous malignant melanoma, in clinical stage I at diagnosis, were studied prospectively to determine the lymph nodal metastatic pattern of the disease, and to find that combination of clinical and pathological variables best predictive of the probability of its occurrence when combined in a linear logistic regression equation based upon a model by Cox. Details of the general pattern of melanoma recurrence are included to provide a necessary background to the nodal metastatic study. Of 66 patients showing melanoma recurrence in 48 (19 males and 29 females) it took the form of lymph nodal metastasis. Of these 50% showed lymph nodal metastasis within 1.1 years of the primary operation and 90% within 3.8 years. Nineteen clinical and pathological variables were tested for association with lymph nodal metastasis, 15 of which showed a significant association and in 7 of these the association was highly significant ($P \leqq 0.0001$). All 19 variables were included in the logistic regression analysis, 6 being selected as providing the best regression 'goodness of fit' and of these 'maximum tumour thickness (Breslow' and 'sex' emerged as the dominant variables. It is concluded that the analysis described provides surgeons, oncologists, and pathologists with a practical method to assess the likelihood in an individual patient of melanoma recurrence to regional lymph nodes. This should enable surgery or other adjunctive therapeutic regimens to be selected at an early stage.

The importance of lymph nodal metastasis to cutaneous malignant melanoma (MM) patients may be considered from the following viewpoints: (1) the development of disseminated disease; (2) treatment; (3) prognosis and (4) survival. With regard to the first and last of these Weidner *et al.* (1976) have stated that in terms of prognosis the early spread of tumour into regional lymph nodes is considered the most critical event. This view was supported by Veronesi *et al.* (1971) who observed that in about 85% of their cases involvement of the regional lymph nodes was an index of the spread of the disease and that metastases beyond the regional nodes had probably occurred but were not detectable. Concerning the second and third of the above points, the provision of an accurate assessment of the probability of nodal recurrence in clinical Stage I patients (tumour still confined to the primary site) shortly following their primary operation would provide the clinician with a means of selecting an optimal therapeutic regimen with the possibility of halting the spread of the disease and inducing cure or a prolonged survival.

Prediction of the probability of lymph nodal metastasis in clinical Stage I MM patients has followed three main lines of study. In the first, selected clinical or pathological variables, either singly or in pairs have been correlated with regional nodal metastasis. For example the work of Clark *et al.* (1969) showed a high degree of correlation between the level of tumour microinvasion according to anatomical landmarks and the incidence of lymph nodal metastases, and also that of Breslow (1975) who showed that the incidence of lymph nodal metastasis from cutaneous MM in clinical Stage I patients was directly proportional to the maximum tumour thickness. In the second approach, illustrated by the work of Schmoeckel & Braun-Falco (1978), a prognostic index was developed based upon the maximum tumour thickness multiplied by the number of mitoses per $mm^2$. In the third, mathematical analyses first introduced by Polk & Linn (1971) and later used in one or other of the various forms of multiple regression analysis (multivariate analysis) were used generally with survival as the dependent variable. An exception is seen in the work of Day *et al.* (1982*a*, *b*) who used a Cox proportional hazard analysis to determine that group of clinical and pathological variables best predictive of MM metastasis to bony or visceral sites.

The present study is a logical continuation of that previously reported by Eastwood & Baker (1983). Its primary aim is to determine by means of a logistic regression analysis, based upon a Cox model, that combination of clinical and pathological variables providing the most reliable estimate of the probability of lymph nodal

Correspondence: J. Eastwood.
Received 21 September 1983; accepted 20 March 1984.

metastasis occurring in a given patient. A secondary aim is to establish the pattern of tumour recurrence with special reference to regional lymph nodal metastasis in the group of patients studied.

### Patients and methods

Clinical and pathological data concerning the 150 patients with cutaneous MM, whose recurrence pattern is described, have already been reported together with the majority of definitions and techniques used (Eastwood & Baker, 1983). Thus only a few additional definitions, histological techniques, and modifications to analytical techniques related to recurrence of the tumour are included here.

### *Patients*

Sixty-six (44%) of the 150 patients studied by Eastwood & Baker (1983) showed recurrent disease and formed the basis of the present work, particular attention being given to those patients showing regional lymph nodal metastasis.

### *Definitions*

With the exception of the lymph drainage area allocated to the popliteal lymph nodes, the definition of regional or first station nodes followed that given in the 'Manual for Staging of Cancer, 1977' (American Joint Committee for Cancer Staging and End-Results Reporting). Concerning the lymph drainage area of the popliteal nodes we have followed Das Gupta & McNeer (1964) and regarded the inguinal nodes as the first station nodes for the lower limb. Clinical staging of patients was according to the M.D. Anderson Hospital system described by Smith (1976) and others.

The following definitions were not included in Part I of this report: (i) a *local recurrence* was one occurring within 3 cm of the primary lesion; (ii) *satellite tumours* were intradermal tumour deposits occurring within the same range, the primary tumour still being present; (iii) *scar recurrences* were tumour deposits occurring along the line of the surgical scar, in the skin graft, or at the graft margins; (iv) *intransit deposits* were tumour deposits occurring more than 3 cm from the primary site in the line of lymphatic drainage; while (v) *central deposits* were tumour deposits at other sites within the body, for example, liver, lung, brain, bone, or disseminated throughout the body. Details concerning the primary treatment of the patients studied and the method of follow-up are given in Part I (Eastwood & Baker, 1983). In essence, unless contraindicated by anatomical or other considerations, primary treatment was surgical and consisted of wide excision of the tumour followed by a skin graft. None of the 66 patients showing lymph nodal metastasis were subject to elective lymphadenectomy or other form of adjunctive therapy in the period prior to nodal recurrence.

### *Histopathology*

Lymphadenectomy specimens were photographed prior to dissection and microscopic examination. Individual lymph nodes were subsequently fixed in 10% formol-saline and were either step-sectioned (small nodes) or a minimum of four blocks were taken from planes parallel to the major axis. Sections were routinely stained with H & E, Goldner's modification of the Masson trichrome stain, the Gomori technique for reticulin, and the Masson–Fontana stain for melanin. All sections were examined by the same histopathologist (J.E.).

### *Statistical methods*

Chi square methods (with Yates' correction when appropriate) were used to test frequency differences in the contingency tables and the association between pairs of variables. Regional nodal metastatic rates were calculated according to the maximum utilization of the life table method (Cutler & Ederer, 1958), the table being modified into a morbidity table by replacement of the table headings "alive at beginning of interval" and "died during interval" by "no nodal metastasis during interval' and "first nodal metastasis during interval". Patients dying without showing nodal metastasis were classified as "lost to follow-up during interval". A stepwise linear regression analysis based upon Cox's (1970) model was used (the reasons for using this model rather than Cox's later (1972) model are stated in an addendum to Part I of this study: see Eastwood & Baker, 1983) the analysis being carried out on the Cyber/720 computer at the University of Bradford using the BMDP PLR program (Engleman, 1979). Of the two variants of the program MLR and ACE, the later was used as previous work had shown that the MLR variant was very expensive in computer time when the number of terms to be analysed was large (>10). The ACE variant is less reliable than the MLR and uses an asymptotic covariance matrix to estimate the regression. It is, however, considerably faster making the computation of large problems practical.

### Results

### *Tumour recurrence*

Sixty-six (44%) of the 150 patients studied showed recurrence of their disease. Details of the sex and

**Table I** Type of first recurrence, patient's sex, interval from primary operation to first recurrence and range of interval in 66 clinical Stage I MM patients at diagnosis

| *Type of recurrence* | *Sex* | *No. of patients (%)* | *Total (%)* | *Mean interval from primary surgery to recurrence (months)* | *Range of interval (months)* |
|---|---|---|---|---|---|
| Scar | M<br>F | 2 (3)<br>7(11) | 9(14) | 25.5 | 2.7– 77.0 |
| Intransits | M<br>F | 1 (1)<br>6 (9) | 7(11) | 18.3 | 3.8– 71.0 |
| Regional lymph nodal | M<br>F | 11(17)<br>23(35) | 34(51) | 23.2 | 3.8–122.0 |
| Distant lymph nodal | M<br>F | 1 (1)<br>0 | 1 (1) | 7.2 | 0.0 |
| Central | M<br>F | 1 (1)<br>4 (6) | 5 (8) | 18.0 | 2.8– 24.8 |
| Simultaneous (two or more) | M<br>F | 4 (6)<br>5 (8) | 9(14) | 31.1 | 3.1– 96.0 |
| Other (e.g. second primary) | M<br>F | 0<br>1 (1) | 1 (1) | 90.1 | 0.0 |
| Grand total | | | 66(100)[a] | | |

[a]Percentages rounded to nearest whole number.

first MM recurrence of these patients are shown in Table I.

*Metastasis to lymph nodes*

At the close of study, in 73% (48/66) of patients showing recurrence it took the form of lymph nodal metastasis, and in 35 of these it provided the first clinical evidence of recurrent disease. Relative to the total numbers of each sex at risk (36 males and 114 females) the proportions were 53% and 25% respectively. The mean age of all patients showing nodal metastasis was 52.9 years (s.d. 16.6) c.f., that of males 55.7 ± 13.1 years; and of females 51.0 ± 18.6 years. The incidence of lymph nodal metastasis by decade and sex is shown in Figure 1. Relative to the total numbers of each sex at risk a male predominance was present in the 5th, 6th, 7th, and 8th decades, and a female predominance in the 3rd, 4th, and 9th.

When related to the number of patients of each sex at risk nodular malignant melanoma (NMM) was superficial spreading melanoma (SSM) accounted for 28% of regional lymph nodal metastasis in males compared to 18% in females.

Table II shows lymph nodal metastasis in

**Figure 1** Age of patient at diagnosis relative to lymph nodal metastasis and sex.

**Table II** Pattern of lymph nodal involvement according to type, patient's sex, mean interval from primary treatment to nodal recurrence and range of interval in 48 MM patients in clinical Stage I at diagnosis.

| *Type of recurrence* | *Sex* | *No. of patients (%)* | *Total (%)* | *Mean interval from primary surgery to recurrence (months)* | *Range of interval (months)* |
|---|---|---|---|---|---|
| Regional nodes only | M<br>F | 12(25)<br>22(46) | 34(71) | 23.3 | 1.7–122.0 |
| Distant nodes only | M<br>F | 1 (2)<br>0 | 1 (2) | 7.2 | 0.0 |
| Nodal metastases preceded by scar recurrence | M<br>F | 0<br>1 (2) | 1 (2) | 64.8 | 0.0 |
| Nodal metastases preceded by transit recurrence | M<br>F | 1 (2)<br>3 (6) | 4 (8) | 21.1 | 8.9– 37.3 |
| Nodal metastases preceded by scar and intransit recurrences | M<br>F | 2 (4)<br>0 | 2 (4) | 14.4 | 7.3– 21.5 |
| Nodal metastases coincident with other forms of recurrence | M<br>F | 3 (6)<br>3 (6) | 6(13) | 27.7 | 3.1– 96.0 |
| Grand total | | | 48(100)[a] | | |

[a]Percentages rounded to nearest whole number.

relation to the general pattern of MM recurrence, the sex of the patients, and the interval and range in months from primary tumour directed surgery to first clinical detection of lymph nodal involvement. Regional lymph nodal metastasis was the form of first recurrence most commonly observed and distal nodal metastasis the least common.

In 34 of the 48 patients showing lymph nodal metastasis recurrent MM had not previously been diagnosed. In 3 (9%) the primary tumour had arisen in the head and neck region, in 5 (15%) in the upper extremity, in 7 (20%) in the trunk region, and in 19 (56%) in the lower extremity. In this last group the primary lesion had been situated between the knee and the foot in 17 (15%) of the patients.

The cumulative proportions of patients showing lymph nodal metastasis are shown in Figure 2. Of all patients developing nodal metastasis 50% did so within 1.1 years of the primary operation and 90% within 3.8 years. The corresponding figures for males were 0.51 years and 1.5 years and for females 2.1 years and >5 years.

Of patients showing lymph nodal metastasis 19 (39%) showed a further recurrence with 11 (23%) showing central MM deposits within a year following lymphadenectomy. The remainder showed other forms of recurrence at intervals of up to nine years following lymphadenectomy and in three patients regional nodal metastasis was followed by further nodal metastasis within 5 years.

**Figure 2** Cumulative incidence of first lymph nodal metastasis in 48 patients (19 males and 29 females) calculated from the time of primary tumour directed surgery. (■) males; (×) females; (●) males and females.

*Associations between single variables and lymph nodal metastasis*

Table III shows 15 variables found to be significantly associated with regional lymph nodal metastasis and lists a further 4 in which a significant degree of association was not reached.

*Logistic regression analysis of variables and probability of lymph nodal metastasis*

Table IV shows the group of predictor variables selected by the ACE variant of the BMDP PLR program as providing the best "goodness of fit" to the logistic regression equation, with lymph nodal

**Table III** Significant associations found between lymph nodal metastasis and 15 of the nineteen potential predictor variables tested.

| *Parameter compared* | $X^2$ | *df* | *P* |
|---|---|---|---|
| Sex of patient | 9.1702 | 1 | <0.01 |
| Site of primary lesion (H & N, UE, T, LE)[a] | 7.9205 | 3 | <0.05 |
| Tumour cross-sectional profile | 19.8183 | 2 | <0.0001 |
| Height above skin surface | 21.5640 | 3 | <0.0001 |
| Maximum tumour diameter (< or > than 10 mm) | 4.3227 | 1 | <0.05 |
| Tumour ulceration | 12.4258 | 2 | <0.002 |
| Maximum tumour thickness (Breslow) | 24.5532 | 3 | <0.0001 |
| Level of microinvasion (Clark) | 18.7750 | 3 | <0.001 |
| Actinic degeneration of dermal collagen | 5.0046 | 1 | <0.05 |
| Tumour cell heterogeneity | 7.8286 | 2 | 0.02 |
| Tumour cell nucleoli (prominent/not prominent) | 6.0407 | 1 | <0.05 |
| Tumour giant cells | 31.2127 | 3 | <0.0001 |
| Tumour cell pleomorphism | 17.4579 | 2 | <0.01 |
| Tumour cell mitotic activity | 20.1610 | 2 | <0.0001 |
| Host reaction (cell) strength | 7.6405 | 2 | <0.05 |

The following parameters were tested but a significant association could not be demonstrated between them and lymph nodal metastasis: age at diagnosis (> or < than 50 years); predominant tumour cell type; vascular invasion; and tumour type (Clark)

[a]H & N—head and neck, UE—upper extremities, T—trunk, LE—lower extremities.

**Table IV** Variables selected for prediction of lymph nodal metastasis, within 5-years of the primary operation, by the BMDP PLR "Ace" stepwise logistic regression program (Engleman 1979)

| *Variable* | *Coefficient* | *S.E.* | *Coeff/S.E.* |
|---|---|---|---|
| Maximum tumour thickness | 0.266 | 0.151 | 1.760 |
| Patient's sex | −2.032 | 0.607 | −3.349 |
| Tumour height above skin surface | | | |
| (1) | 1.230 | 1.062 | 1.159 |
| (2) | 1.263 | 0.653 | 1.935 |
| (3) | −1.549 | 0.657 | −2.358 |
| Tumour cell pleomorphism | | | |
| (1) | 2.175 | 0.850 | 2.558 |
| (2) | −0.104 | 0.537 | −0.193 |
| Tumour cell mitotic acticity | | | |
| (1) | 1.449 | 0.634 | 2.286 |
| (2) | −0.760 | 0.600 | −1.267 |
| Host reaction cell strength | | | |
| (1) | −2.403 | 0.750 | −3.202 |
| (2) | −0.719 | 0.579 | −1.214 |

Goodness of fit $\chi^2 = 48.820$; df = 84; $P$ value = 0.999.

metastasis within five years of the primary tumour directed operation as the dependent variable. Table V shows the estimated correlation matrix in the final model.

Figure 3 shows the percentage of correct classifications of outcome as a function of various points on the scale of computed probabilities.

### Discussion

The characteristic feature of multivariate analysis is the consideration of a set of *n* objects, on each of which are observed the values of *P* variables. The set of objects may be complete or it may be a sample from a larger set. The variables may be continuous or discontinuous, and themselves may be a subset from a larger group (Kendall, 1975). The multivariate techniques, all of which are multiple regression methods, involve a linear function of the independent or prognostic variables. Details of multiple regression techniques are to be found in the work of Armitage (1977) and illustrations of practical applications in that of Lee (1980).

The advantage of multiple regression analysis lies in the fact that the method can be used not only to identify risk factors but also to predict the probability of occurrence of a selected dependent variable, in the present work that of lymph nodal metastasis within a given period of time following primary operation. This probability can also serve as an index of risk. All 6 predictor variables selected by the model show significant association with lymph nodal metastasis and with 3 (maximum tumour thickness, height above skin surface, and tumour cell mitotic activity) the association was highly significant ($P<0.0001$) (Table III). All show a significant association with maximum tumour thickness and there are many significant associations between other pairs within the 6 (see Table II, Eastwood & Baker, 1983).

The purpose of multiple regression analysis, in this study, is to find that combination of variables best predictive of the desired outcome. Day *et al.* (1982*b*) have shown that other combinations of variables may predict the outcome as well or better than the combination selected by the initial Cox regression analysis. This occurs when one or more variables not selected by the primary analysis are correlated with one or more variables selected by that analysis, a situation that occurs in the present study. They refer to this situation as the "alternative model phenomena" and state that it may explain apparent discrepant results obtained by various groups when the Cox proportional hazards analysis is used. Although the earlier Cox model was used in the present study we observed in our earlier work

**Table V** Estimated correlation matrix in final model

| *Variable* | | *1* | *2* | *3* | *4* | *5* | *6* | *7* | *8* | *9* | *10* | *11* |
|---|---|---|---|---|---|---|---|---|---|---|---|---|
| 1. Maximum thickness | | 1.000 | | | | | | | | | | |
| 2. Sex | | −0.531 | 1.000 | | | | | | | | | |
| 3. Tumour elevation | (1) | −0.296 | −0.130 | 1.000 | | | | | | | | |
| 4. | (2) | −0.219 | −0.123 | −0.001 | 1.000 | | | | | | | |
| 5. | (3) | −0.161 | 0.398 | −0.307 | −0.197 | 1.000 | | | | | | |
| 6. Pleomorphism | (1) | 0.208 | −0.294 | −0.176 | 0.157 | −0.167 | 1.000 | | | | | |
| 7. | (2) | −0.442 | −0.054 | 0.264 | 0.038 | −0.226 | −0.193 | 1.000 | | | | |
| 8. Mitoses | (1) | 0.270 | −0.186 | −0.079 | −0.006 | −0.135 | 0.230 | −0.065 | 1.000 | | | |
| 9. | (2) | 0.079 | −0.009 | −0.341 | 0.056 | 0.065 | −0.059 | −0.022 | −0.515 | 1.000 | | |
| 10. Host reaction (C) | (1) | −0.320 | 0.540 | −0.163 | −0.137 | 0.375 | −0.420 | 0.085 | −0.371 | 0.258 | 1.000 | |
| 11. | (2) | −0.176 | 0.257 | −0.109 | −0.191 | 0.189 | 0.11 | 0.050 | −0.086 | −0.196 | −0.212 | 1.000 |

**Figure 3** Percentage of correct classifications at various points on the scale of computed probabilities of outcome relative to patients showing lymph nodal metastasis.

(Eastwood & Baker, 1983) that variants of the BMDP PLR program based upon the 1970 Cox model selected different series of variables as producing the best regression "goodness of fit", a situation that may well have arisen as a result of association between variables in the manner described by Day *et al.* (1982*b*). A knowledge of this possibility is of the greatest importance in seeking an explanation of apparently anomalous results.

The percentage of correct classifications of outcome at various points on the probability scale are shown in Figure 3. Of interest is the rapid fall in accuracy of prediction at the beginning and end of the scale compared with a level of accuracy of about 85% over its greater part. The cause of this is not known but may be the result of a smaller number of patients showing these low and high probabilities of outcome. A graph of this type has importance in that it gives the probability that a given prediction of outcome concerning an individual patient is a correct prediction. It also has the merit that it shows in a simple form whether or not one regression is providing more accurate predictions than another over the whole of the probability scale. The percentage of accurate predictions given at each point on the probability of outcome scale being the final criterion upon which the value of a given regression model of this type can be judged and compared.

The emergence of maximum tumour thickness as the dominant predictor variable in the six selected to form the regression caused little surprise; a correlation between increasing melanoma thickness and risk of regional nodal metastases having been observed by many workers, (e.g. Breslow, 1975 & 1978; Holmes *et al.*, 1976; Balch *et al.*, 1979; and Roses *et al.*, 1982). The choice of sex as the second partial regression coefficient may reflect the marked difference in incidence and rate of lymph nodal metastasis observed between males and females (Table II, Figure 2) with the result that they are forming two significantly different groups in this respect., This view is supported by Weidner *et al.* (1976) who found lymph nodal metastasis to be significantly increased in males, a finding in accord with that of the present study. Of the remaining variables selected as partial regression coefficients, tumour cell mitotic activity showed a high degree of association with lymph nodal metastasis ($P < 0.0001$) and also a high degree of association with maximum tumour thickness (Eastwood & Baker, 1983) suggesting a certain degree of interaction between the two. Likewise, height of the tumour above the skin surface showed a high degree of association with both maximum tumour thickness (Eastwood & Baker, 1983) and with lymph nodal metastasis ($P < 0.0001$). The host reaction (cell) strength and tumour cell pleomorphism were both significantly associated with maximum tumour thickness ($P < 0.05$: Eastwood & Baker, 1983) and with lymph nodal metastasis ($P < 0.05$). A feature that emerges from this study is that the primary variables selected as dominant, namely, tumour thickness and to a lesser extent sex of patient, show concordance with other regression analyses using survival for a given period as the dependent variable.

Details regarding the group of patients forming the study (Tables I, II, and Figure 3) are included to illustrate the clinical and pathological background to this study, to show the relationship of lymph nodal metastasis to other forms of MM

recurrence, and to temporal and other aspects of lymph nodal metastasis. Much of the information included in the section is confirmatory of work done elsewhere and will not, therefore be discussed here.

Finally, it is concluded that the linear logistic regression equation developed in the present work will provide a useful means of assisting the surgeon or oncologist, by providing an index in the form of a probability, relative to an individual patient, of the likelihood of occurrence of regional lymph nodal metastasis. Such a probability can be provided shortly following the primary tumour directed surgery and can give guidance as to the desirability of further surgery e.g. lymphadenectomy, or other form of post-primary adjunctive therapy given at an early stage of the disease when the tumour load is relatively low and maximum effect might reasonably be expected.

Thanks are due to Prof. R.J. Ord-Smith of the Postgraduate School of Studies in Computing, University of Bradford, for constant help and advice relative to computation. We are grateful to Mr. R. Grimshaw, Chief Medical Laboratory Scientific Officer of the Department of Histopathology, Bradford Royal Infirmary, Bradford, and to Mr. P. Harrison, Medical Photographer of St. Luke's Hospital, Bradford, for expert technical assistance. We gratefully acknowledge the help given by the Consultant Surgeons, particularly Mr. T.L. Barclay and Mr. D.J. Crockett, who referred material for examination and allowed access to their clinical notes.

**Appendix** Examples of prognostic forecasts for "A" a patient who showed regional lymph nodal metastases 37.7 months after primary surgery and "B" a patient who was free from all clinical evidence of MM recurrence 9.3 years after primary surgery

| | Covariants and values | Coefficient × variable value | |
|---|---|---|---|
| T. | Maximum tumour thickness | 0.266 × 2.40 | A[a] |
| | | 0.266 × 1.44 | B[b] |
| | *Coefficient × Design variable* | | |
| S. | Patient's sex | | |
| | male | 2.032 | |
| | female | −2.032 | A&B |
| E. | Elevation above skin surface | | |
| | 1. 0.00 mm | −0.944 | |
| | 2. to 1.99 mm | −1.549 | B |
| | 3. 2.00 to 3.99 mm | 1.263 | A |
| | 4. ≧ than 4 mm | 1.230 | |
| P. | Tumour cell pleomorphism | | |
| | 1. mild | 2.071 | |
| | 2. moderate | −0.104 | B |
| | 3. marked | 2.175 | A |
| M. | Tumour cell mitotic activity | | |
| | 1. <1 fig./5 h.p.f. (Gp. 1) | 0.689 | |
| | 2. >1 fig./5 h.p.f. to 1 fig./h.p.f. (Gp. 2) | −0.760 | B |
| | 3. > 1 fig./h.p.f. (Gp. 3) | 1.449 | A |
| H. | Host reaction (cell) strength | | |
| | 1. weak | −3.122 | |
| | 2. moderate | −0.719 | A |
| | 3. strong | −2.403 | B |

[a]The letters "A" and "B" indicate the variant values of patients A and B respectively.

*Calculations*

Patient "A"

$P = e^x/1 + e^x$, where $x = 0.266T + (-2.032) + 1.263 + 2.175 + 1.449 + (-0.719)T$ (maximum thickness) for patient A = 2.40 mm. Therefore $P = 0.94$, thus this patient has a 94% chance of regional lymph nodal metastasis within five years of the primary operation. A similar calculation for patient B indicates that he has a <1% chance of lymph nodal metastasis during the same period.

### References

AMERICAN JOINT COMMITTEE FOR CANCER STAGING AND END-RESULTS REPORTING (1977). *Manual for Staging of Cancer 1977*. Chicago: American Joint Committee.

ARMITAGE, P. (1971). *Statistical Methods in Medical Research*, p. 302, Oxford: Blackwell.

BALCH, C.M., MURAD, T.M., SOONG, S.-J., INGALLS, A.L., RICHARDS, P. & MADDOX, W.A. (1979). Tumor thickness as a guide to surgical management of clinical Stage I melanoma patients. *Cancer*, **43**, 883.

BRESLOW, A. (1975). Tumor thickness. Level of invasion and node dissection in Stage I cutaneous melanoma. *Ann. Surg.*, **182**, 572.

BRESLOW, A., CASCINELLI, N., ESCH, E.P. VAN DER & MORABITO, A. (1978). Stage I melanoma of the limbs: Assessment of prognosis by levels of invasion and maximum thickness. *Tumori*, **64**, 273.

CLARK, W.H. JR., FROM, L., BARNADINO, E.A. & MIHM, M.C. (1969). The histogenesis and biologic behavior of primary human malignant melanomas of the skin. *Cancer Res.*, **29**, 705.

COX, D.R. (1970). *Analysis of Binary Data*, p. 86. London: Chapman & Hall.

COX, D.R. (1972). Regression models and life tables. *J.R. Stat. Soc. Br.*, **34**, 187.

CUTLER & EDERER (1958). Maximum utilization of the life table method in analysing survival. *J. Chron. Dis.*, **8**, 699.

DAS GUPTA, T. & McNEER, G. (1964). The incidence of metastasis to accessible lymph nodes from melanoma of the trunk and extremities. Its therapeutic significance. *Cancer*, **17**, 897.

DAY, C.L. JR., MIHM, M.C. JR., LEW, R.A. & 18 others. (1982*a*). Prognostic factors for patients with clinical Stage I melanoma of intermediate thickness (1.51–3.99 mm). *Ann. Surg.*, **195**, 35.

DAY, C.L. JR., LEW, R.A., MIHM, M.C. JR. & 19 others. (1982*b*). A multivariate analysis of prognostic factors for melanoma patients with lesions $\geqq$3.65 mm in thickness. *Ann. Surg.*, **195**, 44.

EASTWOOD, J. & BAKER, T.G. (1983). Cutaneous malignant melanoma in West Yorkshire: I. A prospective study of variables, survival and prognosis. *Br. J. Cancer*, **48**, 645.

ENGELMAN, L. (1979). BMDP–PLR stepwise logistic regression. In *Biomedical Computer Programs, P. Series*. (Eds. Dixon & Brown). Los Angeles: University of California Press.

HOLMES, E.C., CLARK, W., MORTON, D.L., EILBER, F.R. & BOCHOW, A.J. (1976). Regional lymph node metastases and the level of invasion of melanoma. *Cancer*, **37**, 199.

KENDALL, M. (1975). *Multivariate Analysis*. London: Charles Griffin & Co.

LEE, ELISA T. (1980). *Statistical Methods for Survival Data Analysis*, p. 306. Belmont, California: Lifetime Learning.

POLK, H.C. Jr. & LINN, B.S. (1971). Selective regional lymphadenectomy for melanoma: A mathematical aid to clinical judgement. *Ann. Surg.*, **174**, 402.

ROSES, D.F., HARRIS, M.N., HIDALGO, D., VALENSI, Q.J. & DUBIN, N. (1982). Primary melanoma thickness correlated with regional lymph node metastasis. *Arch. Surg.*, **117**, 921.

SCHMOECKEL, C. & BRAUN-FALCO, O. (1978). Prognostic index and malignant melanoma. *Arch. Dermatol.*, **114**, 871.

SMITH, J.L. JR. (1976). Histopathology and biologic behavior of malignant melanoma. In: *Neoplasms of the Skin and Malignant Melanoma*. p. 293 (Eds. Freitag & Culhane). Chicago: Year Book Medical Publishers.

VERONESI, U., CASCINELLI, N. & PREDA, F. (1971). Prognosis of malignant melanoma according to regional metastases. *Am. J. Roentgenol. Radium Ther. Nucl. Med.*, **111**, 301.

WEIDNER, F., HORNSTEIN, O.P., HERMANEK, P. & WUTZ, G. (1976). Early metastases in regional lymph nodes and prognosis of malignant melanoma. *Arch. Derm. Res.*, **256**, 167.